# Enhancement of Bacterial Survival and Self-Healing Performance in Mortars After Exposure to Negative Temperature Using Alumina Hollow Spheres as Bacterial Carriers

**DOI:** 10.3390/ma18102245

**Published:** 2025-05-12

**Authors:** Yan-Sheng Wang, Yi-Ze Zhou, Xu-Dong Wang, Guang-Zhu Zhang

**Affiliations:** 1 School of Civil Engineering and Transportation, Northeast Forestry University, Harbin 150040, China; 15146025142@nefu.edu.cn (Y.-S.W.); zyz041010@163.com (Y.-Z.Z.); 2 College of Catholic, Songsim Global Campus, The Catholic University of Korea, Bucheon-si 14662, Republic of Korea

**Keywords:** bacteria, alumina hollow spheres, MICP, negative temperature, self-healing ability

## Abstract

Negative temperature environments inhibit bacterial survival in cementitious materials and reduce the self-healing ability of bacteria. To address this challenge, acid-etched alumina hollow spheres are proposed as carriers to encapsulate microorganisms in cementitious materials. The effects of these carriers on the mechanical properties, thermal conductivity, self-healing properties, and self-healing products of specimens after exposure to −20 °C were investigated. Finally, the self-healing mechanism was examined and analyzed. The results demonstrated the effectiveness of the acid-etched hollow microbeads as bacterial carriers. The addition of the alumina hollow spheres participating in the cement hydration reaction enhanced the mechanical properties of the mortar and reduced its thermal conductivity, which supported bacterial survival in the negative temperature environment. Although negative temperature environments may reduce bacterial populations, the hydrolysis of aluminum ions in the alumina hollow spheres during bacterial metabolism resulted in the precipitation of aluminum hydroxide flocs. These flocs adsorbed free calcium carbonate in the pores, converting it into effective calcium carbonate with cementing properties, thus enhancing the crack healing capability of the examined specimens. This microbe-based self-healing strategy, utilizing alumina hollow spheres as bacterial carriers, is anticipated to provide an effective solution for achieving efficient crack self-healing in mortars that is resistant to the detrimental effects of negative temperature conditions.

## 1. Introduction

Self-healing concrete has attracted significant attention from scholars because of its ability to promptly repair cracks, as well as its sustainability and durability [1,2]. Self-healing concrete has the ability to autonomously repair cracks without human intervention, primarily through physical, chemical, and biological repair methods [3]. Physical repair addresses concrete cracks using physical methods and materials without involving chemical reactions. Common approaches include surface sealing, filling, and bonding methods [4]. The chemical repair method uses chemical reactions to repair concrete cracks. Specifically, it involves introducing repair materials with specific chemical properties into concrete cracks. Under certain conditions, these materials react chemically, generating products that ultimately fill the cracks [5]. Common chemical repair methods include osmotic crystallization, chemical grouting [6], and electrodeposition [7]. Compared to physical and chemical repair techniques, biological self-healing offers the advantages of environmental friendliness and cost-effectiveness [8]. The microbial repair method primarily uses the microbially induced calcium carbonate precipitation (MICP) technology for crack self-healing. This method repairs cracks promptly as they form, reducing the repair costs and achieving remarkable healing effects [9,10].

MICP applications in cementitious materials are generally categorized into two types: surface application [11,12,13] and internal incorporation [14,15]. The latter approach has been more widely adopted in practical engineering due to its reduced need for external intervention. The core mechanism of MICP involves bacterial metabolism that generates carbonate ions, which react with calcium ions to precipitate calcium carbonate [16,17,18]. Based on metabolic differences, mineralizing microorganisms are typically classified into three categories, characterized by urease-catalyzed urea decomposition, nitrate reduction, and organic-to-inorganic carbon conversion. Ureolytic strains such as *Bacillus sphaericus* LMG 22557 [19], *Bacillus megaterium* LMG 7127 [20], and *Bacillus licheniformis* DSM 13 [21] have demonstrated effective crack healing capabilities through urea hydrolysis, though the concurrent release of ammonia poses environmental concerns. Denitrifying strains, including *Diaphorobacter nitroreducens* DSM 29460 [22] and *Pseudomonas aeruginosa* DSM 50071 [23], promote CaCO_3_ precipitation via nitrate reduction but produce N_2_O, a potent greenhouse gas, thereby limiting their large-scale application. In contrast, *Bacillus pseudofirmus* DSM 8715 [24] facilitates CaCO_3_ formation through conversion of organic carbon with minimal byproduct generation, offering improved environmental compatibility and attracting increasing research interest in sustainable self-healing technologies.

Khaliq et al. [25] found that after 28 days of healing, the crack healing width of specimens without microorganisms was 125 μm, while that with microorganisms was 380 μm, reflecting a 67.11% improvement due to the incorporation of bacteria. Zhang et al. [26] demonstrated that after 56 days of healing, the crack healing rate of mortar specimens incorporating microbial self-healers directly was approximately 40%. Luo et al. [27] observed that after healing for 20 days, the healing ratio of mortar containing bacteria was 60% higher than that of specimens without bacteria. Collectively, these studies indicated that the direct incorporation of microorganisms leads, however, to low bacterial survival rates, mainly because of the high alkalinity and densification in the concrete structure. To overcome this challenge, researchers have suggested and used various materials and methods to protect the incorporated microorganisms.

Porous materials are commonly selected as encapsulation carriers for microorganisms in concrete to alleviate the compressive stress on microorganisms during cement hydration and to mitigate the effects of the highly alkaline environment. Mignon et al. [28] found that after 28 days of healing, 32% of the cracks were healed, and the water permeability of the specimens was reduced by 80% when Bacillus cereus encapsulated in hydrogel was used as a carrier in concrete. Rauf et al. [29] reported that natural fibers, when used as microbial carriers, effectively repaired cracks with a max width of 800 μm and improved the crack healing rate by 50%. Wiktor et al. [30] found that when microorganisms were immobilized on expanded clay particles and incorporated into mortar specimens, cracks with a max width of 460 μm on the specimen surface were completely healed at a healing age of 100 days. Additionally, materials such as zeolite [31], diatomite [32], and expanded perlite [33] have been studied as potential microbial carriers for incorporation into concrete to maintain microbial viability.

In recent years, researchers have explored various vectors for encapsulating microorganisms, focusing on improving repair rates at ambient healing temperatures. Additionally, increasing the low-temperature resistance of microbial self-healing agents has garnered attention, as current research indicates that microbial survival is adversely affected by subzero temperatures. Yan et al. [34] reported that the crack healing width of mortar specimens containing biomass self-healing agents increased by 63.6% after conditioning at −20 °C compared to that of specimens without self-healing agents. Cacchio et al. [35] observed that under low-temperature conditions (4 °C), mortar specimens doped with bacteria produced both surface and internal crack-healing products. However, the time required for complete crack healing was longer compared to that in ambient conditions (25 °C). Novitsky et al. [36] discovered that the rate of calcium carbonate precipitation at cracks in mortar specimens doped with bacteria gradually increased as the maintenance temperature rose from 2 °C to 32 °C. Therefore, to enhance the application of microbial self-healing concrete in low-temperature regions, the adverse effects of subzero temperature conditions on MICP technology must be addressed.

This study focuses on the adaptability of the microbially induced calcite precipitation (MICP) technology under subzero temperatures, aiming to enhance the survival and mineralization efficiency of bacteria during crack repair in cold regions. To achieve this, a novel approach was adopted by utilizing acid-etched alumina hollow spheres as carriers for encapsulating and immobilizing bacteria, thereby constructing a self-healing system with low-temperature protective capabilities. The study systematically investigated the impact of the carrier on bacterial healing performance under two curing conditions, i.e., 25 °C and −20 °C, followed by healing in a 30 °C environment after crack formation. The healing effectiveness was validated through mechanical property testing, crack healing characterization (using ultrasonic pulse velocity (UPV) tests and microscopic imaging), and microstructural analysis techniques, including scanning electron microscopy–energy dispersive X-ray spectroscopy (SEM-EDS), X-ray diffraction (XRD), and thermal conductivity measurements. The results showed that the alumina carrier facilitated the release of Al^3+^ ions via hydrolysis, promoting the deposition of small-sized CaCO_3_ crystals and accelerating the early-stage crack closure. Additionally, its thermal resistance properties helped mitigate the inhibitory effect of low temperatures on bacterial metabolism, enabling complete crack closure during the healing stage in specimens cured at subzero temperatures. This study proposes the innovative use of acid-etched alumina spheres as carriers to enhance the MICP healing mechanism in cold regions, offering a new pathway for the engineering application of the MICP technology under extreme climatic conditions.

## 2. Materials and Methods

### 2.1. Acid-Modified Alumina Hollow Spheres

The alumina hollow spheres used in this study were sourced from Anhui Juteng New Material Technology Co., Ltd., Chuzhou, China and a NH_4_F/HCl solution was used as the etching reagent, prepared by mixing 500 mL of 1.2 mol/L ammonium fluoride (NH_4_F) and 500 mL of 1 mol/L hydrochloric acid (HCl). Specifically, the alumina hollow spheres were immersed in hydrofluoric acid for 12 h and mixed using a magnetic stirrer at 120 rpm for 30 min every 2.5 h to enhance etching uniformity and effectiveness [37]. The acid-etched alumina hollow spheres were then neutralized with a 1 mol/L calcium hydroxide solution and adjusted to pH 7, monitoring the acidity with pH paper. The modified alumina hollow spheres were removed, dried, and weighed until reaching a constant weight. The test protocol was based on ASTM C128-15 [38], which is a standard method for assessing the specific gravity and absorption of fine aggregates. According to previous studies [39] the water uptake behavior of porous materials typically follows a two-phase trend, characterized by rapid initial absorption followed by stabilization at saturation. Based on this understanding, water absorption was measured using the tea bag method at selected time points (1, 5, 10, 20, 30, 60, 120, 720, and 1440 min) to capture the full absorption profile. As shown in Table 1, the water absorption of alumina hollow spheres increased from 47.3% to 70.2% after acid etching, confirming that the hydrofluoric acid etching method effectively enhanced water absorption.

**Figure 1:**
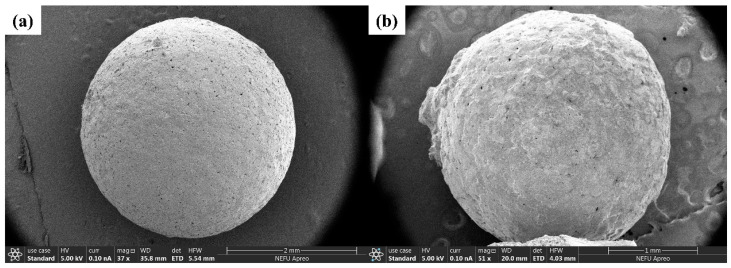
SEM images showing the microstructure of alumina hollow spheres: (**a**) before acid etching; (**b**) after acid etching.

Figure 1a presents the SEM image of the as-received alumina spheres, showing a smooth and dense surface structure with diameter primarily ranging from 80 to 200 μm. Figure 1b shows the spheres after acid etching, where partial dissolution of the outer dense layer reveals an inner porous shell structure, with pore sizes of approximately 100–200 nm. These structural features provide a favorable architecture for capillary-driven nutrient absorption and bacterial encapsulation.

### 2.2. Self-Healing Agent Preparation

**Figure 2:**
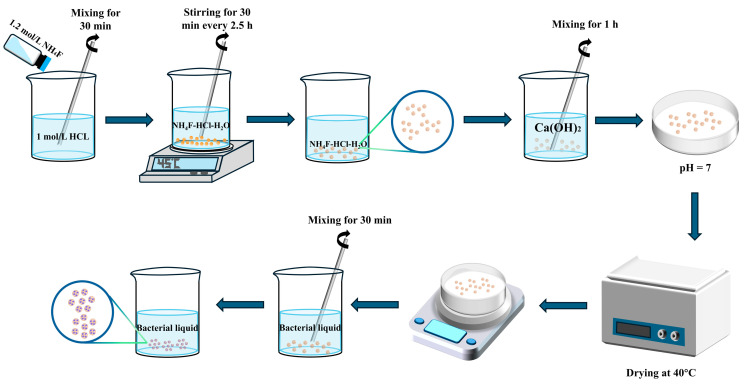
Preparation process of bacterial self-healing agent.

The strain employed was *Bacillus pseudofirmus* DSM 8715, obtained from the China Center of Industrial Culture Collection (CICC), Beijing, China. The literature reports that under sufficient moisture and oxygen, the spores of *Bacillus pseudofirmus* can germinate into vegetative cells and aerobically metabolize calcium lactate. This process releases CO_2_, which subsequently hydrates to form HCO^3−^ and reacts with Ca^2+^ in an alkaline environment to precipitate CaCO_3_, enabling biomineralization and crack sealing within a cementitious matrix [40]. A spore suspension was prepared following a method that we previously described [41]. The detailed procedure (Figure 2) is as follows: a liquid medium was prepared according to the formulation in Table 2; subsequently, a sterile 1 mol/L sodium carbonate solution was added to adjust the pH of the medium; the bacteria were then inoculated into the liquid medium and incubated in a constant-temperature shaker at 27 °C for 24 h, with a shaking speed of 120 rpm; and the bacterial suspension was then centrifuged at 5000 rpm. To satisfy the experimental requirements, the spore precipitate was diluted with distilled water to an OD600 of 0.6, which corresponded to a bacterial concentration of approximately 4.0 × 10^9^ CFU/mL. To minimize the risk of background microbial contamination, all preparation and mixing steps were conducted under sterile conditions. Specifically, the operations were carried out within a laminar flow hood, and all instruments were surface-sterilized with 75% ethanol prior to use.

### 2.3. Mortar Specimen Ratios and Preparation

The materials used for mortar proportioning included ordinary Portland cement (OPC), standard sand, tap water, the bacterial suspension, calcium lactate, and the alumina hollow spheres. Prior to use, 60 g of acid-etched hollow alumina spheres were immersed in 45 mL of bacterial suspension (OD_600_ = 0.6, corresponding to approximately 4.0 × 10^9^ CFU/mL [42]) for 30 minutes. After 24 hours of static incubation, the initial spore loading was estimated to be approximately 3 × 10^9^ CFU per gram of carrier. During mixing and early curing, the absorbed liquid was gradually released from the hollow spheres into the matrix, thereby contributing to the cement hydration process. To ensure comparability, the total water-to-cement ratio (w/c), including both free water and released absorbed water [43], was uniformly controlled to be 0.5 across all groups. The chemical composition of the cement is presented in Table 3. Previous studies have indicated that optimal strength is achieved when calcium lactate is used as a nutrient at a 1 wt.% of the cement [44]. Therefore, 1 wt.% of calcium lactate was used. Three groups of mortars were prepared according to the mix proportions in Table 4, labeled as Control, B, and AB. The Control group consisted of water, cement, and sand. Group B included water, the bacterial suspension, cement, and sand, while Group AB contained the healing agent (microorganisms incorporated in acid-etched alumina hollow spheres), water, cement, and sand. To evaluate the healing ability of the bacterial self-healing agent in mortar specimens under ambient and subzero temperature conditions, the specimens were first cured at 25 °C for 28 days and then exposed to 25 °C or −20 °C for another 28 days.

### 2.4. Crack Preparation and Healing Method

**Figure 3:**
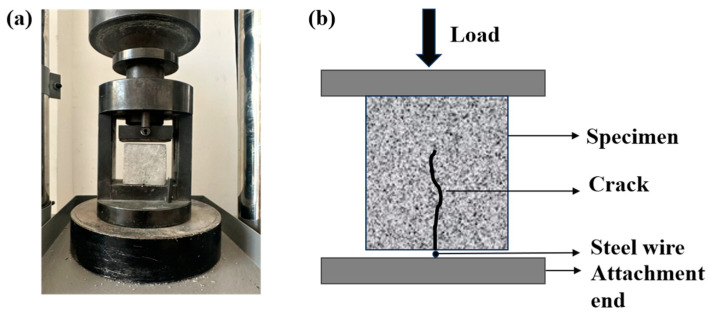
Crack preparation device: (**a**) equipment setup; (**b**) schematic diagram.

Cracks were created in 50 mm cube specimens using an automatic cement flexural and compressive testing machine (DYE-300S, Wuxi Dejiayi Testing Instrument Co., Ltd., Wuxi, China), resulting in visible cracks on the specimen surfaces. The equipment used for crack pre-fabrication is shown in Figure 3. For crack pre-fabrication, a wire strip with a diameter of approximately 1 mm was placed beneath the specimen, and a loading rate of 2 kN/s was applied. This resulted in the formation of visible cracks on the surface of the specimen. To assess the healing ability of mortar specimens with varying crack widths, the specimens were classified into the following groups based on crack width: <400, 400–450, 450–500, and >500 μm. After prefabrication, the specimens were water-cured at 30 ± 1 °C and oxygenated for 30 min daily until the target healing age was reached.

### 2.5. Test Procedure

#### 2.5.1. Thermal Conductivity

The thermal conductivity of the mortar specimens was measured at a curing age of 28 days using a laser heat conductivity testing instrument (LAF467 HyperFlash, Netzsch, Selb, Germany). The tests were conducted at the temperatures of 25 °C and −20 °C. The instrument operated at an acquisition rate of 2 MHz, with a laser voltage of 250 V and a pulse width of 0.6 ms. Its measurement ranges were 0.01–10,001 mm²/s for the thermal diffusivity coefficient and 0.1–2000 W/(m·K) for thermal conductivity. The samples, with dimensions of 10 mm × 10 mm × 30 mm, were collected from both the Control group and the alumina-containing mortar group. The surface of each sample was polished with sandpaper and coated with a uniform layer of graphite for testing. Each measurement was performed three times at each temperature, and the average value was recorded.

#### 2.5.2. Mechanical Properties Test

Compressive strength tests were conducted on 50 mm × 50 mm × 50 mm cubic specimens at the curing ages of 7, 28, and 56 days using a universal testing machine, following ASTM C109-21 [45]. A loading rate of 2400 N/s was applied until complete failure. Flexural strength was measured on 40 mm × 40 mm × 160 mm prism specimens in accordance with ASTM C293-16 [46], using a loading rate of 50 N/s until complete fracture. For each group, three specimens were tested, and the average value and standard deviation were calculated for further analysis.

#### 2.5.3. Measurement of Crack Filling

The closure of the cracks in the mortar specimens during the healing process was monitored using an optical microscope (Olympus SZX10, Olympus Corporation, Tokyo, Japan), which was equipped with a Leica illumination system, Wetzlar, Germany. The “Measure Tool” in Adobe Photoshop 21.1.0.106^®^ was employed in combination with the measurement module of OlympusImageJ 2.4.1 to quantify the width (Wt) and the area of the cracks (St) at designated healing ages (0, 7, 28, and 56 days). The average crack Wt was calculated by measuring the widths of three cracks along their lengths. The crack area healing rate (*η*) and crack width healing rate (*R*) were calculated using Equations (1) and (2), respectively

(1) $$\eta =\frac{S_{t}-S_{0}}{S_{0}}\times 100\%$$

(2) $$R=\frac{W_{t}-W_{0}}{W_{0}}\times 100\%$$

*S*_0_: initial crack area;

*S*_*t*_: crack area at time t (days);

*W*_0_: initial crack width;

*W*_*t*_: crack width at time t (days).

#### 2.5.4. Scanning Electron Microscopy (SEM) Test

The healing products on the crack surfaces of the mortar specimens were harvested at 56 days of healing for SEM analysis to examine the micro-morphological features of the healing products. The samples were polished and dried in an oven at 50 °C for 48 h. Before SEM analysis, the samples were coated with platinum using a magnetron-type coater.

#### 2.5.5. X-Ray Diffraction (XRD) Test

To analyze the crystal types of the self-healing products, XRD analysis (XRD-7000, Shimadzu, Japan) was performed on deposits collected from the cracks of the mortar specimens at 56 days of healing. Healing deposits were scraped from the crack surfaces using a scalpel, ground manually with a grinding bowl, and sieved through a 320-mesh sieve for XRD analysis. The XRD analysis was conducted using a Cu Kα radiation source (λ = 1.5406 Å), over a 2θ range of 5–80° with a step size of 0.02°, and the time per step was 1 s. 

#### 2.5.6. Ultrasonic Pulse Velocity (UPV) Test

**Figure 4:**
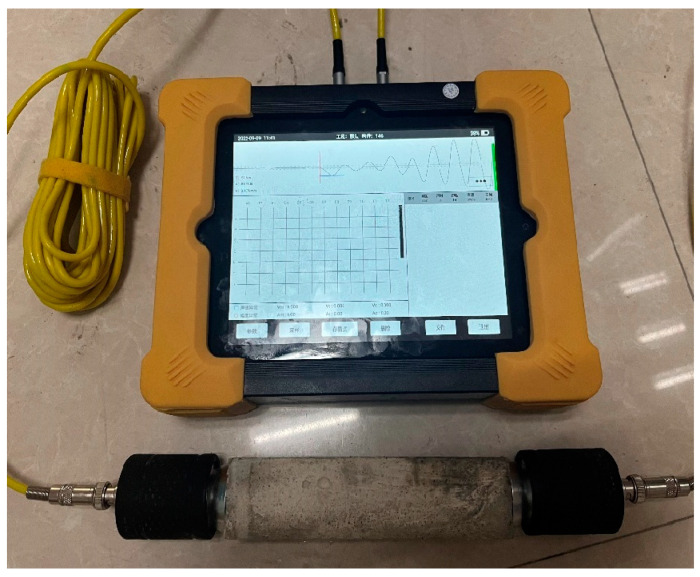
UPV testing instrument.

A concrete crack defect comprehensive tester was used to evaluate the crack healing effects. The specimen size was 40 mm × 40 mm × 160 mm. The principle of the UPV test is to measure the time required for a longitudinal stress wave to pass through a matrix of 160 mm length (Figure 4). The probes were placed in contact with the surface of the mortar specimen, and the wave propagation time was recorded with an accuracy of at least 0.1 μs. The UPV was measured by applying couplant to the planar transducers and pressing it against the specimen surface at both ends. Three independent specimens were prepared for each group, and the average values and standard deviations were calculated for statistical analysis.

**Figure 5:**
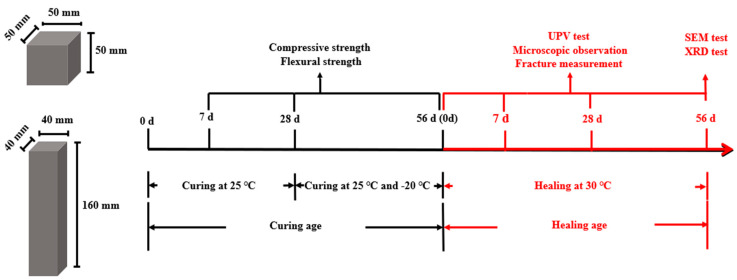
The specific process of the experimental tests.

As shown in Figure 5, two specimen types were used, as follows: 50 mm × 50 mm × 50 mm cubes for compressive testing, and 40 mm × 40 mm × 160 mm prisms for flexural testing. All specimens were initially cured under standard conditions (20 ± 2 °C, RH > 96%) for 28 days, then transferred to either 25 °C or −20 °C environments for an additional 28 days. Their mechanical properties were tested at 7, 28, and 56 days of curing. Subsequently, cracks were introduced, and all samples were transferred to a 30 °C environment to initiate the healing process. Healing was assessed at 0, 7, 28, and 56 days using UPV testing and microscopic crack imaging. At 56 days of healing, SEM and XRD analyses were performed to link macroscopic recovery with microstructural evolution. All specimens were cured and healed under constant temperature conditions (e.g., −20 °C or 25 °C), and all tests were conducted immediately upon reaching the designated curing or healing age. This static temperature setup was deliberately chosen to eliminate uncertainty from temperature fluctuations and ensure experimental consistency. Therefore, no temperature ramping or crossover periods were applied during the testing phase.

## 3. Results and Discussion

### 3.1. Mechanical Properties

**Figure 6:**
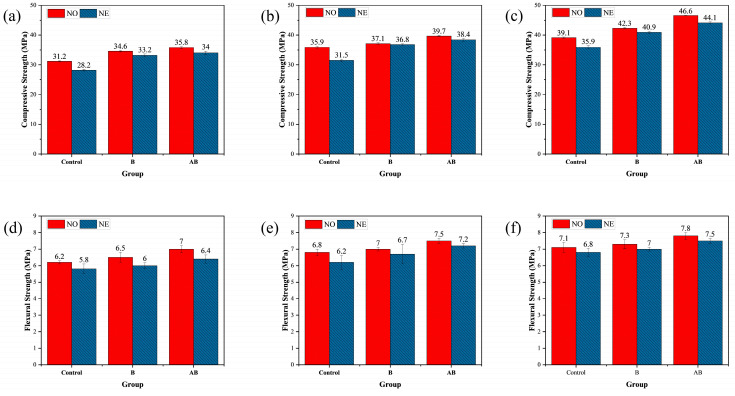
Mechanical properties of specimens at different curing ages: (**a**) 7 days, compressive strength; (**b**) 28 days, compressive strength; (**c**) 56 days, compressive strength; (**d**) 7 days, flexural strength; (**e**) 28 days, flexural strength; (**f**) 56 days, flexural strength.

Figure 6 illustrates the effects of the self-healing agent on the compressive and flexural strengths of the mortar specimens at the curing ages of 7, 28, and 56 days. At 7 days of curing (25 °C), the compressive strengths of NO-B and NO-AB increased by 10.90% and 14.74%, respectively, compared to that of NO-Control. This improvement was primarily attributed to the germination of the bacterial spores in the cement matrix, which metabolically produced CaCO_3_ that filled some of the pores in the mortar specimens, thereby enhancing the compressive strength. The additional 3.47% increase in compressive strength of the NO-AB group compared to the NO-B group was attributed to the inclusion of the etched alumina hollow spheres as bacterial carriers in the cement matrix. These spheres participated in the hydration reaction, forming calcium aluminate, which further enhanced the compressive strength [47]. At 28 and 56 days of curing (25 °C), the compressive strength of the NO-B group increased by 9.92% and 8.18%, respectively, compared to that of the NO-Control group. The compressive strength of the NO-AB group increased by 6.60% and 10.16%, respectively, compared to that of the NO-B group. Regarding the specimens exposed to the negative temperature, the 56 days compressive strength of the NE-Control group decreased by 9.52% compared to that of the NO-Control group. This reduction was attributed to the negative temperature environment, which slowed the hydration process of the cement and reduced the generation of hydration products [48]. The compressive strengths of the NE-B and NE-AB groups decreased by 3.42% and 5.67%, respectively, compared to those of the NO-B and NO-AB groups. This decrease was attributed to the inhibition of cement hydration and reduced microbial survival under negative temperature conditions, where some bacteria were inactivated. Consequently, the generation of deposits decreased, limiting the pore-filling effect and reducing the compressive strength [49]. Nevertheless, the compressive strength of the NE-AB group increased by 7.26% compared to that of the NE-B group. Additionally, the low thermal conductivity of the alumina hollow spheres effectively protected bacterial survival under negative temperature conditions, ultimately enhancing the compressive strength of the NE-AB group [47].

Analyzing the flexural strength data showed that the strength of the mortar specimens in the NO-B and NO-AB groups increased by 4.8% and 12.9%, respectively, compared to that of the NO-Control group at 7 days of curing. At 28 days of curing, the trend of flexural strength was similar to that observed at 7 days. As previously discussed, the incorporated bacterial spores germinated into bacteria, metabolizing calcium lactate to produce CaCO_3_, which filled the internal pores and enhanced the flexural strength. Additionally, calcium aluminate, produced by the etched alumina hollow spheres used as carriers, further contributed to the increase in flexural strength [47]. At 28 days of curing, the trend of flexural strength was similar to that observed at 7 days. The flexural strength of the mortar specimens in the NO-B group increased by 5.33% compared to that of the NO-Control group. The flexural strength of the mortar specimens in the NO-AB group increased by 3.8% compared to that of the NO-B group. Compared to the specimens cured at 25 °C (NO-Control, NO-B, and NO-AB), the flexural strength of all three groups cured at the negative temperature decreased by 4.2%, 4.1%, and 4.3%, respectively. Consistent with the compressive strength findings, the negative temperature hindered cement hydration [50]. Furthermore, the negative temperature conditions caused mechanical damage to some microbial cells due to water crystallization in the pores, reducing the microbial survival rate. This led to the formation of fewer healing products during the healing phase and weakened the pore-filling effect [49]. The 7.1% increase in flexural strength of the NE-AB specimens compared to the NE-B specimens was attributed to the participation of the alumina hollow spheres in the cement hydration reaction and their role in protecting bacterial survival under negative temperature conditions.

### 3.2. Thermal Conductivity of the Mortars

The thermal conductivity of mortar specimens is influenced by multiple factors, including porosity, chemical composition, component proportions, and crystalline structure [51]. In our case, the incorporation of alumina hollow spheres contributed to a reduction in thermal conductivity through two primary mechanisms: (1) the inherently complex crystalline structure of alumina introduced a tortuous path for phonon transport, impeding heat conduction; and (2) the hollow internal structure of the spheres reduced the effective solid-phase continuity within the matrix, thereby further decreasing the heat transfer rate. Table 5 presents the thermal conductivity test results for the Control and AB specimens at the test temperatures of −20 °C and 25 °C. The thermal conductivity of the Control and AB specimens at −20 °C was 2.817 W/(m·K) and 2.4 W/(m·K), respectively. This confirmed that the addition of alumina effectively reduced the thermal conductivity of the mortar specimens. This reduction was primarily attributed to the complex crystal structure of alumina, which complicated the heat transfer pathway and impeded the heat flow [50]. Additionally, the hollow structure of the alumina spheres decreased the rate of heat conduction in the mortar matrix, further reducing the thermal conductivity. The thermal conductivity of the Control and AB specimens at 25 °C was 2.618 W/(m·K) and 2.307 W/(m·K), respectively, indicating that the thermal conductivity of the AB specimens was further reduced as the test temperature increased.

### 3.3. Self-Healing Properties

**Figure 7:**
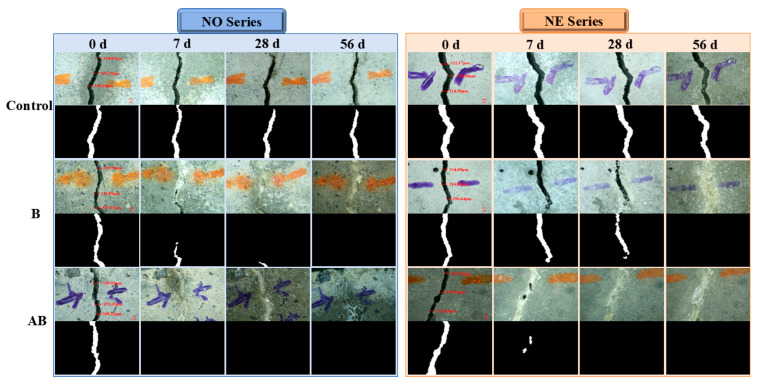
Images of healed cracks at 0, 7, 28, and 56 days.

**Figure 8:**
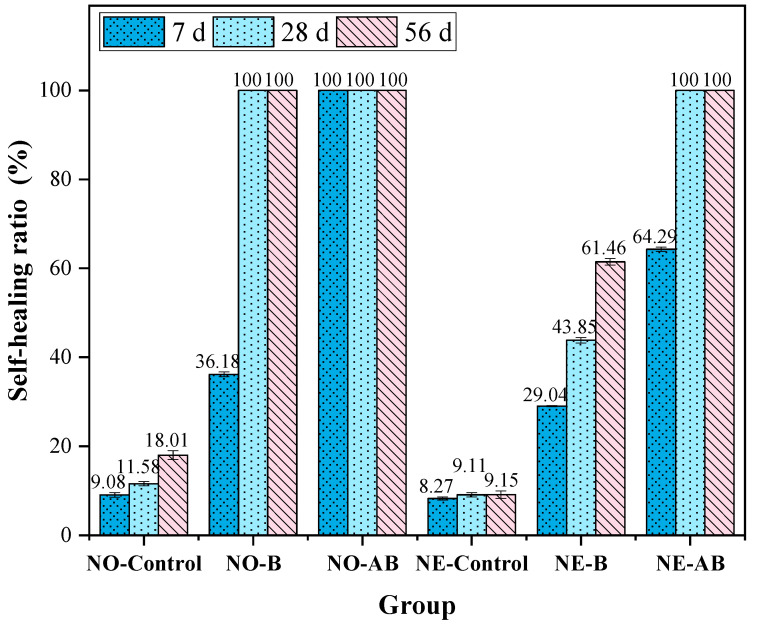
Self-healing ratios of cracks with different widths at healing ages of 7, 28, and 56 days.

The healing of each group was observed at 0, 7, 28, and 56 days of healing age at 30 °C. The crack healing results of the six groups of mortar specimens are presented as healed area values (Table 6), images of healed cracks (Figure 7), and self-healing ratio (Figure 8). At 7 days of healing age, the healing rate of the NO-Control specimens was 9.08%, primarily due to the hydration reaction of cement filling the cracks. In comparison, the healing rates of the NO-B and NO-AB specimens were 36.18% and 100%, respectively. This outcome was due to the addition of the self-healing agent to the cement matrix, which promoted bacterial spore germination and the subsequent metabolic production of CaCO_3_, which effectively filled the cracks. The NO-AB specimens achieved complete healing at an early age, not only due to bacterial metabolism but also due to the accelerated closure of the cracks facilitated by the formation of calcium aluminate crystals from the interaction between the modified alumina hollow spheres and the cement matrix [47]. Additionally, the SEM tests confirmed that the hydrolysis of aluminum ions in the carrier precipitated aluminum hydroxide flocs, which promoted the deposition of small-sized CaCO_3_ crystals. The NO-B specimens also achieved complete healing at 28 days of healing. Compared to the NO-Control group, the healing rate of the NO-B specimens was increased by 88.42%.

The 28-day healing rate of the specimens after exposure to negative temperature curing revealed a 49.19% reduction in the healing rate of the NE-Control mortar compared to the NO-Control group. As mentioned earlier, the healing of the Control group specimens primarily relied on cement hydration, which was adversely affected by the negative temperature environment [50]. The healing rate of the NE-B specimens decreased by 38.54% compared to that of the NO-B group. This decrease was attributed to mechanical damage to some microbial cells caused by water crystallization in the pores under negative temperature conditions, which reduced the microbial survival rate and ultimately limited deposit generation and healing efficiency [49]. However, the addition of the etched alumina hollow spheres as a bacterial carrier in the cement matrix led to the complete closure of the cracks in the NE-AB specimens. This improvement was primarily attributed to the low thermal conductivity of the specimens under extreme temperature conditions, which favored bacterial survival. Additionally, the SEM analysis confirmed that the hydrolysis of aluminum ions in the carriers resulted in the formation of aluminum hydroxide flocs, which facilitated the precipitation of small-sized calcium carbonate crystals. After 56 days of healing, only the NO-B mortar specimens achieved complete healing. In contrast, the NE-B group lacked the protective effect of the alumina hollow spheres, leaving the bacteria vulnerable to the extreme temperature, which resulted in a final healing rate of only 61.46%.

During the mixing process, calcium lactate was uniformly incorporated into the mortar matrix as a nutrient source. Acid etching generated porous structures on the surface of the hollow alumina spheres (see Figure 1b), allowing calcium lactate in the pore solution to diffuse spontaneously into the carrier via capillary action [52,53]. Upon crack formation, moisture and oxygen ingressed the mortar, triggering the germination of dormant spores into metabolically active vegetative cells. These cells aerobically metabolized calcium lactate, producing CO_2_, which reacted with water to generate HCO_3_^−^. In the highly alkaline environment (pH > 12), HCO_3_^−^ combined with free Ca^2+^ ions to form CaCO_3_, thereby enabling microbial-induced crack healing through biomineralization.

**Figure 9:**
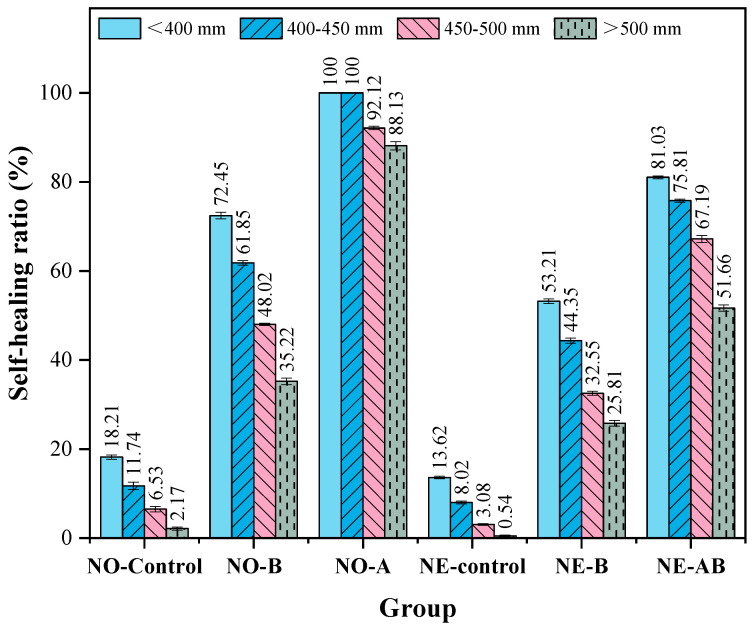
Self-healing ratios of cracks with varying widths.

The healing of cracks with varying initial widths (<400, 400–450, 450–500, and >500 μm) was evaluated. The experimental data are presented in Table 7, and the healing rates for different crack widths were calculated using Equation (1) and are plotted in Figure 9. For cracks of less than 400 μm in width, the healing rate of the NO-Control group was 18.21%, attributed to the limited capacity of cement hydration to fill the cracks. The healing rate of NO-B was 72.45%, demonstrating the effectiveness of bacterial self-healing agents in repairing cracks within this range. The complete closure of the cracks in NO-AB indicated that the incorporation of modified alumina further enhanced bacterial healing, achieving complete closure of cracks of less than 400 μm. For the NE series specimens, the healing rate of the NE-Control group decreased to 13.62%, as the negative temperature conditions retarded the cement hydration reaction [50]. The healing rate of the NE-B specimens decreased to 53.21%, as the negative temperature conditions, as discussed earlier, caused mechanical damage to some microbial cells. This reduced the survival of effective bacteria during the healing period, ultimately lowering the amount of healing deposits. The healing rate of the NE-AB specimens decreased to 81.03%.

For cracks with widths of 400–500 μm, the healing rate of the NO-Control specimens decreased to 6.53–11.74%, while the healing rates of NO-B and NO-AB were 48.02–61.85% and 92.12–100%, respectively. Compared to the NO-B specimens, the NO-AB specimens demonstrated superior healing performance, confirming that the addition of alumina enhanced bacterial effectiveness in healing cracks within the 400–500 μm range and maintained a high healing efficiency. For the NE series specimens, the healing rate of the NE-Control group further decreased to 3.08–8.02%, while the healing rates of the NE-B and NE-AB specimens declined to 32.55–44.35% and 67.19–75.81%, respectively. For cracks > 500 μm, the healing rates of the NO-B and NO-AB specimens were 35.22% and 88.13%, respectively. The addition of the carrier ensured that cracks larger than 500 μm maintained a high healing capacity after curing at room temperature. After exposure to negative temperature conditions, the healing rates of the NE-B and NE-AB specimens declined to 25.81% and 51.66%, respectively. For cracks > 500 μm, the negative temperature environment significantly reduced the healing efficiency in both groups of specimens due to its destructive effects. The negative temperature conditions were shown to have a greater destructive impact on the microbial cells than the healing promotion effect provided by alumina hollow spheres, making cracks larger than 500 μm difficult to heal.

### 3.4. UPV Test

**Figure 10:**
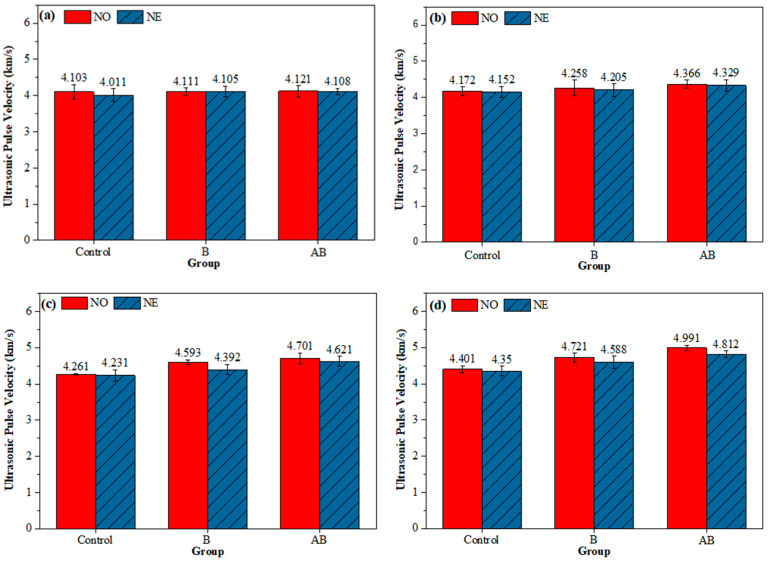
UPV of healed specimens: (**a**) 0 days; (**b**) 7 days; (**c**) 28 days; (**d**) 56 days.

Figure 10 illustrates the UPV values of the specimens at 0, 7, 28, and 56 days of healing age, which were used to indirectly evaluate the healing performance of the mortar specimens. The UPV of the three groups of mortar specimens cured at two temperature conditions followed the same trend, i.e., AB > B > Control at 7, 28, and 56 days. Specifically, the UPV values of the NO-Control specimens increased by 6.78%, while those of the NO-B group increased by 14.84%, which was attributed to bacterial metabolism producing CaCO_3_ that partially filled the cracks and enhanced the UPV. The UPV of the NO-AB specimens increased by 21.11%, which was attributed to the involvement of the etched alumina hollow spheres in cement hydration reactions, producing calcium aluminates that filled pores and cracks and promoting MICP, which ultimately enhanced UPV [47,54]. The UPV of the NO-Control specimens increased by 2.28% compared to that of the NE-Control group, which was attributed to the negative temperature conditions that hindered the cement hydration process [50]. The UPV of the NE-B and NE-AB mortar specimens decreased by 2.90% and 3.72%, respectively, compared to that of the NO-B and NO-AB specimens. This decrease was attributed to the negative temperature conditions causing mechanical damage to the microbial cells through water crystallization in the pores, reducing microbial survival and the production of deposits [49]. Additionally, the UPV of the NE-AB specimen increased by 4.88% compared to that of the NE-B specimen, which was attributed to the inclusion of the alumina hollow spheres, which lowered the mortar’s thermal conductivity, enhancing bacterial survival at the applied negative temperature, and participated in the cement hydration reaction [47].

### 3.5. Microscopic Analysis

#### 3.5.1. SEM Test

**Figure 11:**
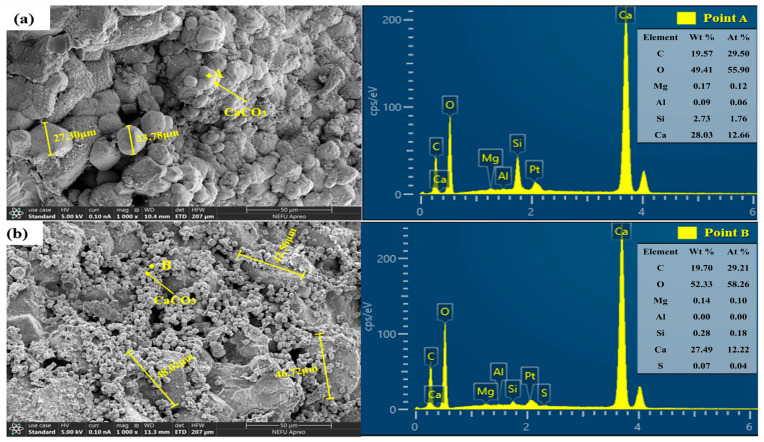

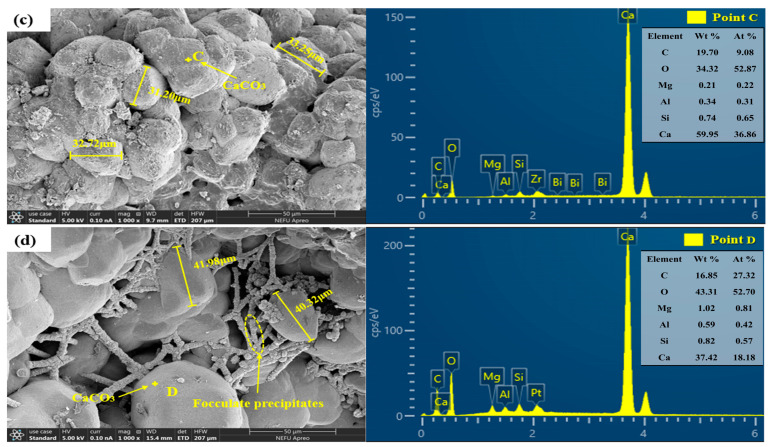
SEM images of healing products at 56 days of healing age: (**a**) NO-B; (**b**) NO-AB; (**c**) NE-B; (**d**) NE-AB.

SEM was used to analyze the micro-morphological characteristics of the healing products in mortars at 56 days of healing. The elemental compositions were confirmed through EDS analysis. As the NO-Control and NE-Control groups exhibited limited crack closure effects, with healing rates below 15%, and primarily relied on cement hydration products to fill the cracks, SEM observations were not conducted for these two groups. Figure 11 presents the SEM-EDS images of the healing products for NO-B, NE-B, NO-AB, and NE-AB. For the mortar specimens with direct addition of the self-healing agent (NO-B and NE-B groups), Figure 11a,c reveal that the healing products at the cracks primarily consisted of CaCO_3_. The healing products in the NO-B specimens were more complete and denser than those in the NE-B group, primarily due to the negative temperature reducing the number of viable bacteria, adversely affecting the amount and morphology of the CaCO_3_ healing products. The CaCO_3_ crystals at the cracks of the NO-AB specimens (Figure 11b) were larger and featured numerous small cubic crystals attached to their surfaces, confirmed as calcium carbonate through EDS analysis. This phenomenon was primarily attributed to the hydrolysis of aluminum ions in the hollow spheres, within the carrier, resulting in the precipitation of aluminum hydroxide flocs with strong adsorption properties [55]. The flocs precipitated on the surfaces of CaCO_3_ crystals and within the pores, adsorbing free CaCO_3_ and converting it into “effective” calcium carbonate with a cementing effect. This densified the CaCO_3_ crystal structure and significantly enhanced the healing capacity of the specimens. Cubic crystals with irregular crystal structure were attached to the surfaces, along with a substantial amount of flocculent material connecting the CaCO_3_ crystals. The reduced number of small-sized CaCO_3_ crystals and their irregular crystal structure in the NE-AB specimens compared to the NO-AB group were primarily attributed to the negative temperature conditions, which diminished the effective bacterial population. This reduction led to a lower amount of free CaCO_3_ in the pores during the healing phase, thereby weakening the ability of the aluminum hydroxide flocs to precipitate CaCO_3_ crystals. The CaCO_3_ crystals in the healing products were observed to be larger in the mortar specimens doped with the alumina hollow spheres (NO-AB and NE-AB groups) compared to those without the alumina hollow spheres (NO-B and NE-B groups). This was attributed to the effective protective role of the alumina hollow spheres as carriers for bacterial self-healing agents, which preserved a greater number of viable microorganisms. These microorganisms synergistically produced calcium carbonate crystals, favoring the formation of larger healing products [56]. As shown in Figure 11c, flocculent precipitates were clearly observed, identified by EDS analysis as aluminum hydroxide, which likely formed via hydrolysis of Al^3+^ ions released from the acid-etched alumina carriers. In contrast, Figure 11d shows minimal evidence of CaCO_3_ deposition, indicating that bacterial activity was significantly suppressed at subzero temperatures, resulting in insufficient carbonate generation. These observations suggest that, even under low-temperature conditions where microbe-induced healing is inhibited, alumina-based carriers may assist in physical crack blocking through Al(OH)_3_ precipitation, thereby contributing to early-stage sealing performance.

#### 3.5.2. XRD Test

**Figure 12:**
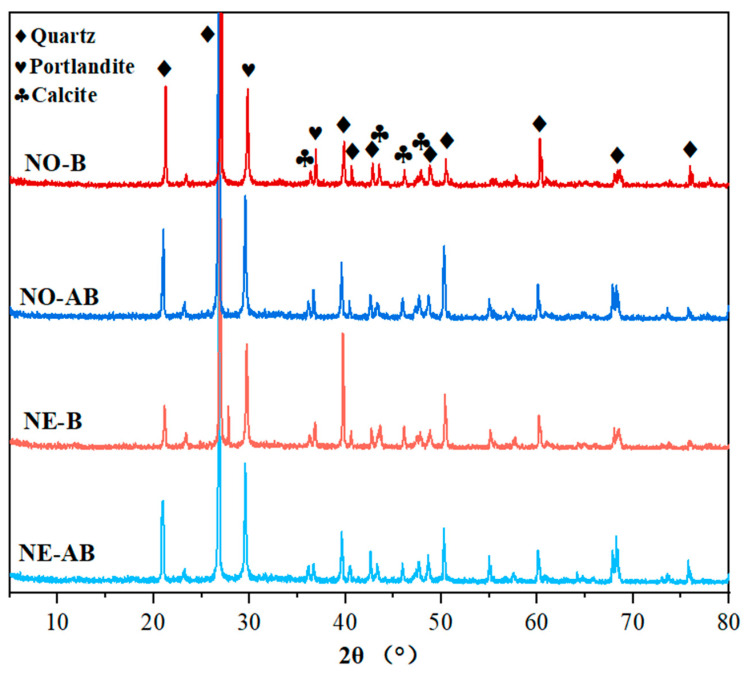
XRD results for healing products in cracks of specimens at 56 days of healing.

Figure 12 presents the XRD results of the healing products collected from the cracks at 56 days of healing age. The mortar specimens from the NO-B, NE-B, NO-AB, and NE-AB groups exhibited major peaks at 2θ values at 29.5°, corresponding to calcite (ICDD #05-0586) peaks. Meanwhile, the XRD results also exhibited pronounced characteristic peaks for quartz (ICDD #046-1045) and Ca(OH)₂ (ICDD #073-7671). The intensity of the calcite characteristic peaks was higher for the specimens doped with the alumina hollow spheres and cemented with bacteria (NO-AB and NE-AB) compared to those with the direct addition of the bacterial self-healing agent (NO-B and NE-B). This is consistent with the higher number of CaCO_3_ crystals observed at the cracks in the NO-AB and NE-AB specimens in the SEM observations. Specifically, aluminum ions in the alumina carrier generated aluminum hydroxide flocculates, which adsorbed free calcium carbonate in the pores, causing it to precipitate and generate a greater number of small CaCO_3_ crystals, thus enhancing the intensity of the characteristic calcite peak. As temperature influenced bacterial survival and subsequently affected the amount of calcium carbonate crystals generated, the intensity of the calcite characteristic peaks was higher in the NO series specimens (NO-B and NO-AB) than in the NE series specimens (NE-B and NE-AB).

## 4. Conclusions

In this study, a microbial self-healing agent was prepared using acid-etched alumina hollow spheres as bacterial carriers, and the self-healing effect at 30 °C was investigated in specimens that cracked after first being cured at −20 °C for 28 days. To clarify the effect of adding the microbial self-healing agent on the healing pattern of the cracks, cracks with different initial widths were introduced, and their healing rates were observed. The healing mechanism was further investigated through microscopic tests. The main conclusions are as follows:

1. Alumina hollow spheres as bacterial carriers enhanced mortar strength by generating calcium aluminate via hydration and protecting bacteria under a negative temperature through low thermal conductivity, with flexural strength trending similarly to compressive strength.
2. Thermal conductivity tests evaluated the thermal properties of the Control specimens and those incorporating the alumina hollow spheres at −20 °C and 25 °C. The results showed that the addition of the alumina hollow spheres reduced the thermal conductivity of the specimens by 14.8% at −20 °C and 11.9% at 25 °C, respectively. This confirmed that the complex crystal structure of the hollow spheres disrupted heat conduction pathways, while their hollow architecture decreased the heat transfer rates within the matrix. These combined mechanisms mitigated the temperature sensitivity of the concrete.
3. Crack healing tests showed that the alumina hollow spheres enhanced bacterial self-healing. The specimens with the self-healing agent cured at 25 °C achieved full crack closure within 7 days. At −20 °C, the healing efficiency of the bacteria-only specimens dropped to 61.46%, but full healing occurred with the alumina hollow sphere carriers. Note: cracks > 500 μm could not fully close, as the negative temperature-induced microbial cell damage overwhelmed the carrier’s protective effect.
4. SEM revealed numerous small cubic CaCO_3_ crystals on deposition surfaces, attributed to aluminum ion hydrolysis from the alumina hollow spheres, which generated aluminum hydroxide flocs that induced free CaCO_3_ precipitation. The carriers also supported bacterial survival, promoting synergistic CaCO_3_ crystal growth.
5. XRD confirmed that the alumina hollow spheres enhanced the intensity of the calcite peaks observed for the NO-AB and NE-AB specimens. Aluminum ion hydrolysis produced aluminum hydroxide flocs that adsorbed free calcium carbonate, promoting crystal formation. The higher calcite peak intensity in the NO-series specimens (NO-B/NO-AB) was linked to temperature-dependent bacterial survival affecting CaCO_3_ yield.

Future work will focus on optimizing carrier formulations and encapsulation processes to enhance microbial survivability and mineralization activity in extreme climates, advancing the application of MICP-based self-healing technologies in cold-region infrastructure.

## Figures and Tables

**Table 1:** Water absorption rates of modified and unmodified alumina hollow spheres.

Time (min)		1	5	10	20	30	60	120	720	1440
Water absorption rate (%)	Modified	2	5.1	13.4	22.1	35.0	42.6	47.3	47.3	47.3
	Unmodified	4	8.2	20.5	46.7	56.6	68.7	70.2	70.2	70.2

**Table 2:** Composition of liquid medium [41].

Component	Peptone (Biochemistry)	Beef Extract	NaCl	Deionized Water	MnSO_4_
Content	3 g/L	3 g/L	15 g/L	1 L	0.005 g/L

**Table 3:** Chemical composition of cement.

Material	Composition (wt%)								
	CaO	SiO_2_	Al_2_O_3_	SO_3_	Fe_2_O_3_	MgO	K_2_O	Others	Loss on Ignition
Cement	65.10	19.52	4.37	3.21	3.22	1.01	0.86	0.80	1.91

**Table 4:** Mix proportions of mortars.

Group	Cement/g	Fine Aggregates		Water/g	Bacteria/mL	Calcium Lactate/g
		Standard Sand/g	Modified Alumina Hollow Spheres/g			
Control	1000	2000	-	500	-	-
B		2000	-	460	40	10
AB		1940	60	460	40	10

**Table 5:** Thermal conductivity of specimens.

Test Temperature °C	Flash Points	Control Group			AB Group		
		Thermal Diffusion Coefficient mm^2^/s	Thermal Conductivity W/(m·K)	Specific Heat Capacity Cp.J/(g·K)	Thermal Diffusion Coefficient mm^2^/s	Thermal Conductivity W/(m·K)	Specific Heat Capacity Cp.J/(g·K)
−20 °C	Point 1	1.841	2.816	0.764	1.592	2.407	0.757
	Point 2	1.851	2.829	0.769	1.582	2.392	0.753
	Point 3	1.834	2.805	0.760	1.588	2.401	0.758
	Average value	1.842	2.817	0.764	1.587	2.400	0.756
25 °C	Point 1	1.448	2.607	0.914	1.278	2.293	0.905
	Point 2	1.458	2.625	0.901	1.288	2.311	0.899
	Point 3	1.456	2.622	0.885	1.292	2.318	0.888
	Average value	1.454	2.618	0.900	1.286	2.307	0.897

**Table 6:** Crack-healed areas (mm^2^).

Groups	Healing Age			
	0d	7d	28d	56d
NO-Control	1.244	1.131	1.100	1.02
NO-B	1.921	1.226	0	0
NO-AB	1.465	0	0	0
NE-Control	2.152	1.974	1.956	1.955
NE-B	1.243	0.882	0.698	0.479
NE-AB	1.398	0.798	0	0

**Table 7:** Average initial crack width and average 90-day healed crack width for each group of specimens.

Crack Width/(μm)	NO-Control		NO-B		NO-AB		NE-Control		NE-B		NE-AB	
	0d	90d	0d	90d	0d	90d	0d	90d	0d	90d	0d	90d
<400	386.23	314.78	371.38	0	366.12	0	389.10	335.21	398.91	184.93	368.05	0
400–450	420.28	371.32	431.04	0	429.33	0	441.01	405.73	410.72	228.69	435.28	0
450–500	472.98	442.05	459.66	0	471.87	0	462.93	448.07	482.05	324.66	490.17	33.14
>500	535.68	524.97	563.17	65.79	522.06	9.76	584.28	581.12	598.97	447.25	576.25	61.49

## Data Availability

The original contributions presented in this study are included in the article. Further inquiries can be directed to the corresponding authors.

## Abbreviations

The following abbreviations are used in this manuscript:

MICP	Microbially induced carbonate precipitation
SEM-EDS	Scanning electron microscopy and energy-dispersive X-ray spectroscopy
XRD	X-ray diffraction
UPV	Ultrasonic pulse velocity
NO	Normal temperature
NE	Negative temperature
NO-Control	Control specimens cured at normal temperature
NO-B	Specimens incorporating only bacteria and cured at normal temperature
NO-AB	Specimens incorporating both alumina hollow spheres and bacteria and cured atnormal temperature
NE-Control	Control specimens cured at negative temperature
NE-B	Specimens incorporating only bacteria and cured at negative temperature
NE-AB	Specimens incorporating both alumina hollow spheres and bacteria and cured atnegative temperature
